# Outcome and EBRA migration analysis of a reconstruction cage in acetabular revision arthroplasty: a clinical and radiological study

**DOI:** 10.1007/s00402-020-03722-x

**Published:** 2020-12-22

**Authors:** Dietmar Dammerer, Philipp Blum, David Putzer, Annelies van Beeck, Michael Nogler, Martin Thaler

**Affiliations:** 1 Department of Orthopaedics and Traumatology, Medical University of Innsbruck, Anichstrasse 35, 6020 Innsbruck, Austria; 2 Department of Experimental Orthopedics, Medical University of Innsbruck, Innrain 36, 6020 Innsbruck, Austria; 3 Antwerp University Hospital, Wilrijkstraat 10, 2650 Edegem, Belgium

**Keywords:** Cup migration, Total hip arthroplasty, Cup revision, Restoration cup, Einzel-Bild-Röntgen-Analyse (EBRA)

## Abstract

**Purpose:**

Restoration cages and bone allografts have been proposed to manage severe acetabular bone defects. We aimed to investigate the migration behaviour of a restoration cup and impacted allograft bone in severe acetabular defects with Einzel-Bild-Röntgen-Analyse (EBRA).

**Methods:**

Applying a retrospective study design, 64 cases treated between 2009 and 2016 were reviewed. We determined the preoperative Charlson Comorbidity Index (CCI), pre- to postoperative WOMAC score, blood loss and functional outcome. From preoperative x rays, the acetabular deficiencies were classified according to Paprosky. Cup migration analyses were performed with EBRA.

**Results:**

Mean age at surgery was 73 (range: 38–93) years. According to the classification by Paprosky et al., 50% (*n* = 32) of our patients showed a type III B and 28.1% (*n* = 18) a type III A defect. Radiological follow-up for migration analysis was 35 (range: 4–95) months. Migration analysis showed a mean cup migration of 0.7 mm (range: 5.7–9.6) medial and 1.8 mm (range: 1.7–12.6) cranial.

**Conclusion:**

In conclusion, acetabular restoration cages in combination with bone impaction grafting showed a low revision rate at a mean follow-up of 35 months. Mean cup migration revealed low rates after 2 years and suggested a stable postoperative implant position.

## Introduction

As the number of patients receiving total hip arthroplasty (THA) grows, the number of cases requiring revision surgery increases accordingly [1]. The rate of acetabular cup revision is gradually rising and there is a heightened risk for osteolysis, prosthesis loosening over time and an increase in life expectancy [2].

Patients with failed acetabular cup revision surgery frequently have severe bone cavities and segmented defects resulting in pelvic deficiency, and present a difficult situation for revision arthroplasty [3]. Specifically, to restore the pelvic bone stock, the acetabular component should be placed in the correct anatomical position and the joint stability optimized [4]. The reconstruction should permit stable fixation of the new acetabular component and should lead to the restoration of the center of rotation [5, 6].

The current literature shows that substantial bone loss has been a major concern in revision THA [3, 7]. Bone impaction grafting has proven to be a helpful method for restoring the bone stock [8]. In cases of severe acetabular defects the use of a so-called restoration cage allows the bony acetabular defect to be bridged and protected until a bone stock has been formed [9]. The literature reports the use of restoration cages to be a valuable option in treating severe bone defects [3, 5, 10]. Although, migration analysis is a well-established method for predicting life expectancy and long-term outcome of implants in primary THA [11, 12], data regarding migration analysis in acetabular reconstruction cages are still missing. Accurate measurement of early migration of revision acetabular components is crucial to establish whether surgical techniques and implant designs are evaluated for their long-term outcome [13].

For acetabular reconstructions, reinforcement rings or restoration cages are frequently used. However, the clinical and radiological results of restoration cages are controversial [3, 5, 10]. A small number of previously published studies have reported implant success rates ranging from 58% to 100% [3, 5, 10]. None of those studies investigated the migration behavior of restoration cages.

The purpose of our study was to investigate the clinical outcome and perform a migration analysis of a frequently used acetabular restoration cage in revision arthroplasty of the acetabulum.

## Materials and methods

The study protocol was approved by the Ethics committee of the Medical University of Innsbruck, Austria, Europe. Written informed consent was obtained from all subjects before participation. All methods and measurements were carried out in accordance with relevant guidelines and regulations.

Applying a retrospective study design with a prospective follow-up, we reviewed medical histories of all consecutive patients who received a Graft Augmentation Prosthesis (GAP) II by Stryker Orthopaedics (Mahwah, NJ, USA) at our Department between September 2009 and August 2016. The GAP II is an acetabular restoration cage and consists of a sand-blasted titanium shell with a superior-posterior lip and an inferior hook, which is used for fixation at the Köhler teardrop. Two superior plates allow screw fixation to the ilium, and screw fixation of the titanium shell to the pelvic bone is possible [3, 5].

During the above-mentioned period, 64 GAP II cages were implanted in 60 patients (female = 37; male = 23). In three patients the GAP II was implanted bilaterally, while one patient was revised on the same side with a GAP II after the initial GAP II prosthesis failed. Sociodemographic and surgical data are shown in Table 1.

**Table 1:** Sociodemographic and surgical data of the study group. The range is given in brackets

Number of patients	Female	37
	Male	23
	Total	60
Side	Left	33
	Right	31
	Total	64
Mean age at surgery (years)		73 (38–93)
Body Mass Index (kg/m^2^)		26.3 (18.4–44.4)
WOMAC Score (total)	Preoperative	58.5 (26.1–82.9)
	Postoperative	30.8 (11.2–79.2)
Surgical indication	Aseptic cup loosening	32
	Infection	10
	Periprosthetic acetabulum fracture	7
	Polyethylene wear	5
	Acetabular protrusion of former cup and aseptic loosening	3
	Cup malposition	1
	Material and periprosthetic fracture	1
	Hip dysplasia and secondary osteoarthritis	1
	Osteoarthritis and severe acetabular defect	1
	Periprosthetic femur fracture	1
	Femoral neck fracture and acetabular defect	1
	Femoral head necrosis and acetabular defect	1
Operation time [min] (range)		163 (81–332)
Surgical approach	Direct anterior	58
	Lateral transgluteal	5
	Dorsal	1
Surgical position	Supine	63
	Lateral	1
Calculated Blood loss [ml]		2348 (346–4816)
Classification acetabular defect Paprosky	Type I	2
	Type II A	1
	Type II B	8
	Type II C	3
	Type III A	18
	Type III B	32

Indications for revision surgery were aseptic cup loosening (*n* = 32), infection (*n* = 10), periprosthetic acetabulum fracture (*n* = 7), polyethylene wear (*n* = 5), protrusio of the implanted acetabular component (*n* = 3), cup malposition (*n* = 1), material and periprosthetic fracture (*n* = 1), severe hip dysplasia and secondary osteoarthritis (*n* = 1), osteoarthritis with acetabular defect (*n* = 1), periprosthetic fracture of the femur (*n* = 1) and femoral neck fracture with acetabular defect (*n* = 1). The GAP II was used once as a primary implant in a femoral head necrosis, resulting in an acetabular bone defect (*n* = 1).

From preoperative X-rays the acetabular deficiencies were classified according to Paprosky et al. [14]. Accordingly, 3.1% (*n* = 2) showed type I, 1.6% (*n* = 1) type II A, 12.5% (*n* = 8) type II B, 4.7% type II C (*n* = 3), 28.1% type III A (*n* = 18) and 50% type III B (*n* = 32) (Table 1).

Two senior consultant surgeons performed over 90% of all the surgeries mentioned in this study. Three different approaches were used: in 58 cases a direct anterior approach [15, 16], in five cases a lateral transgluteal approach [17] and once a dorsal approach [18, 19]. The surgical approach was chosen in way of a) initial approach, b) the bone defect, which has to be addressed in revision surgery and c) the reachability through the chosen approach. The majority of the procedures were performed in the supine position (*n* = 63). In one case the surgery was performed in lateral decubitus.

The range of motion was pre- and postoperatively assessed with reference to the medical histories. In addition, patients were asked to complete the Western Ontario and McMaster Universities Osteoarthritis Index (WOMAC) questionnaire pre- and postoperatively [20].

The patients were followed with radiographs before discharge, six weeks after surgery and 12 months postoperatively. Additional radiographs were performed if the patient had any complaints after revision THA. All radiographs were taken in standardized conditions with the exact same technique (anterior–posterior (AP) radiographs; patient standing in the upright position and full weight-bearing) following the EBRA protocol [21, 22]. The postoperative radiographs were used to evaluate the ingrowth of the bone graft and assess the position of the hook, which is mentioned for fixation around the Köhler teardrop. Based on the radiological criteria for implant failure described by Sembrano et al. [23], a stable cup was defined as the presence of intact hooks, screws and plates, the absence of increased radiolucency in the area of the implant and an implant migration of less than 5 mm at the time of the last follow-up. According to the criteria of Sloof et al. [24], an additional radiological analysis of the bone graft was performed to evaluate the consolidation of the bone graft. This was defined as the presence of trabecular bone crossing the graft-host junction.

**Fig. 1:**
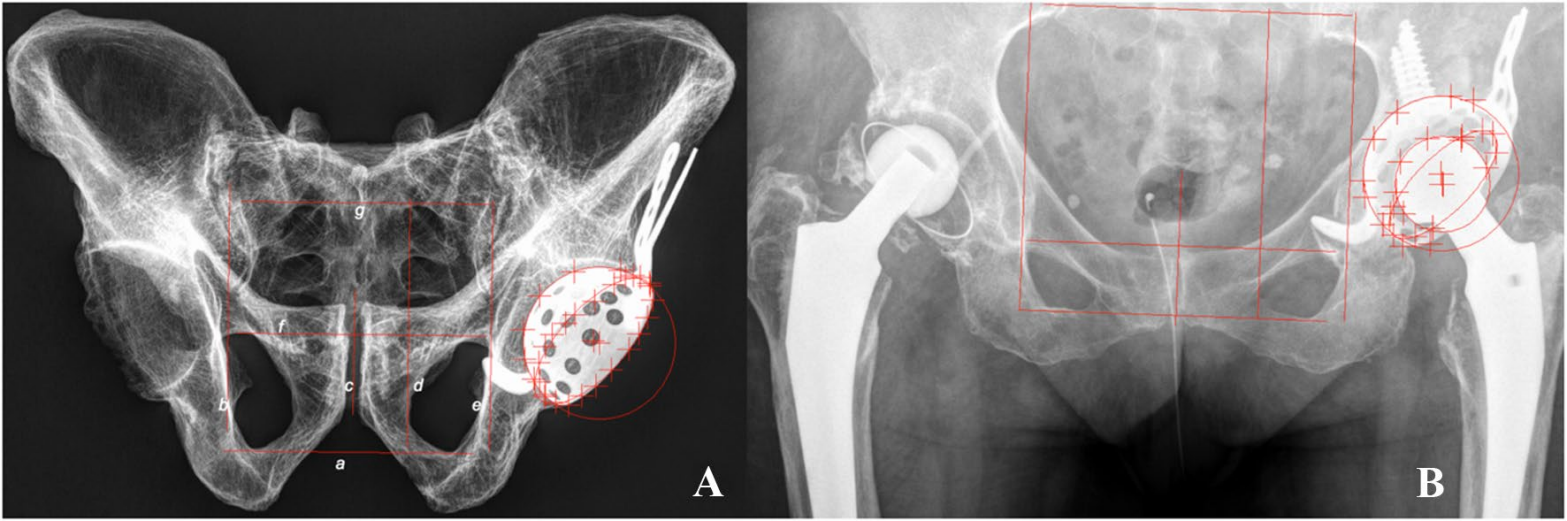
Anterior-posterior x rays of a pelvic bone as follows: **a** anatomical phantom and **b** patient showing a GAP II cage and drawn tangents for migration analysis with EBRA, (a) base line, (b) right line, (c) symphysis line, (d) foramen line, (e) left line and (g) top line

Before migration analysis, we validated EBRA in revision reconstruction cases. We took more than 30 × rays following the EBRA protocol and in different pelvic tilt positions. EBRA migration analysis was performed with a comparability limit of 3.0 mm (95% confidence interval). Negative horizontal migration values were defined as medial migration. Negative vertical migration (distal migration) up to 1 mm was due to the limited accuracy of the EBRA measurement method. Based on the pre-test results and together with the EBRA engineers, the measurement results were able to be validated and verified. Figure 1 shows the anatomical phantom and a patient case including EBRA reference.

Einzel-Bild-Röntgen-Analyse (EBRA) was used to measure migration of the GAP II restoration cage. Included were all patients with at least four postoperative plain hip x rays. EBRA is a well-established method that evaluates standard anterior–posterior radiographs without requiring additional means at exposure (e.g., ball markers). Simulating the spatial situation, it computes parameters of longitudinal and transverse migration of the prosthetic cup and femoral head. A comparability algorithm using a grid of transverse and longitudinal tangents of the pelvis contour divides serial radiographs into sets of comparable ones. Migration is measured only between comparable radiographs. The 95% confidence limits for EBRA results are 1.0 mm for longitudinal and 0.8 mm for transverse migration [21, 25]. EBRA migration analysis was performed [21, 25] by an independent investigator, who was not involved in the surgeries or postoperative treatment of the patients.

## Statistical analysis

An independent statistician performed the statistical analyses. For statistical analysis, Excel (Microsoft Office Professional Plus 10, Redmond, WA, USA) and Graph Pad Prism (Version 7.0, GraphPad Software Inc., La Jolla, CA, USA) were used. A descriptive statistic was calculated for all parameters, including mean, median, standard deviation, interquartile range, minimum, maximum and range. An analysis of variance, performed for repeated measurements, tested the results of the migration analysis for significant differences (*p* < 0.05). In addition, a Kaplan–Meier survival analysis was performed for the implant.

## Results

**Fig. 2:**
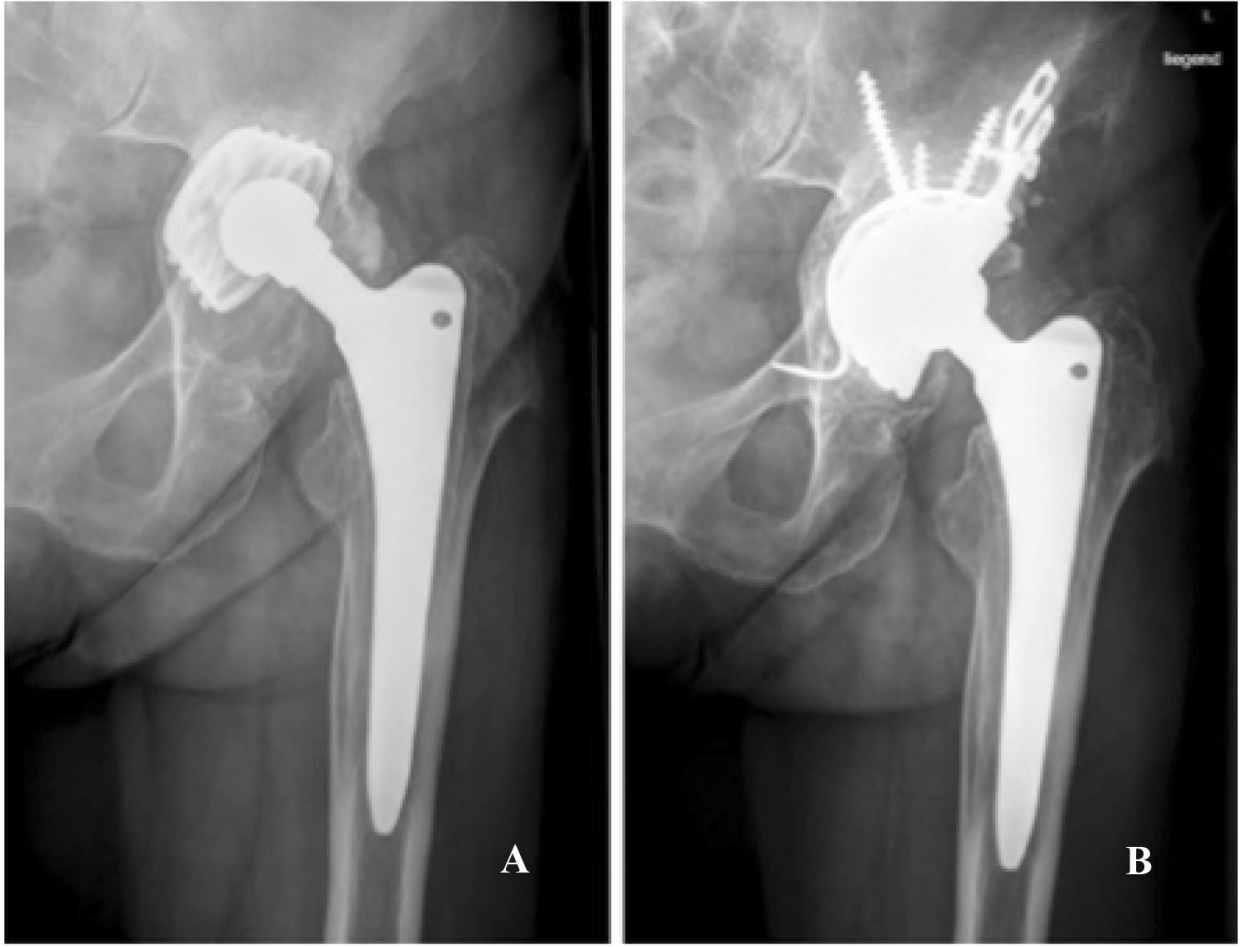
Preoperative radiograph of a Paprosky type III B defect (**a**). 35 months after surgery, stable position of the GAP II restoration cage and good consolidation of the bone graft can be observed (**b**)

The mean age at surgery was 73 (range: 38–93) years. Mean Body Mass Index was 26.3 (range: 18.4–44.4) kg/m2. Mean clinical follow-up was 29 (range: 1–96) months. Mean radiological follow-up for the EBRA migration analysis was 35 (range: 4–95) months. Average surgery time was 163 (range: 81–332) min (Table 1). According to the bone impaction grafting technique, allogenic cancellous bony chips were used in 62 of the 64 hips. The bone grafts were taken from the Department’s own bone bank. Consolidation of the bone graft was observed in 59 of the 62 cases at the final radiological follow-up (Fig. 2).

In pre- and postoperative comparison we documented an improvement in the mean WOMAC score from 58.5 (range: 26.1–82.9) to 30.8 (range: 11.2–79.2). Estimated blood loss was calculated using the formula by Mercuriali [26]. Each substitution of concentrated red blood cells, administered intra- or postoperatively until the fifth day, was included in the calculation with a quantity of 280 ml and a haematocrit of 0.54. Calculated blood loss averaged 2348 ml (± 1037 ml; range: 346–4816 ml). Three patients died 11, 22 and 42 months after implantation of the GAP II cage. None of the causes of death were related to the surgery. Further details are shown in Table 2.

**Table 2:** Cohort demographics for all used cages, allogenic bone, screws, cups and results of the pre- and postoperative compares of the WOMAC score

Allogenic bone (*n*)		62 (97%)
Allogenic bone consolidation (*n*)		59 (92%)
Screws used (mean; *n*)		5 (range; 3–7)
Mesh used (*n*)		13 (20.3%)
Diameter and size restoration cage (*n*)	72 mm	5 (7.8%)
	68 mm	3 (4.7%)
	64 mm	11 (17.2%)
	60 mm	18 (28.1%)
	56 mm	21 (32.8%)
	54 mm	1 (1.5%)
	52 mm	4 (6.2%)
	50 mm	1 (1.5%)
Cemented cup in cage (*n*)	Müller II^®^	43 (67.2%)
	Avantage^®^	21 (32.8%)
WOMAC score (mean, total)	Preoperative	58.5 (range; 26.1–82.9)
	Postoperative	30.8 (range; 11.2–79.2)
Leg length discrepancy (mean; cm)		1 (range; − 2–3)

**Fig. 3:**
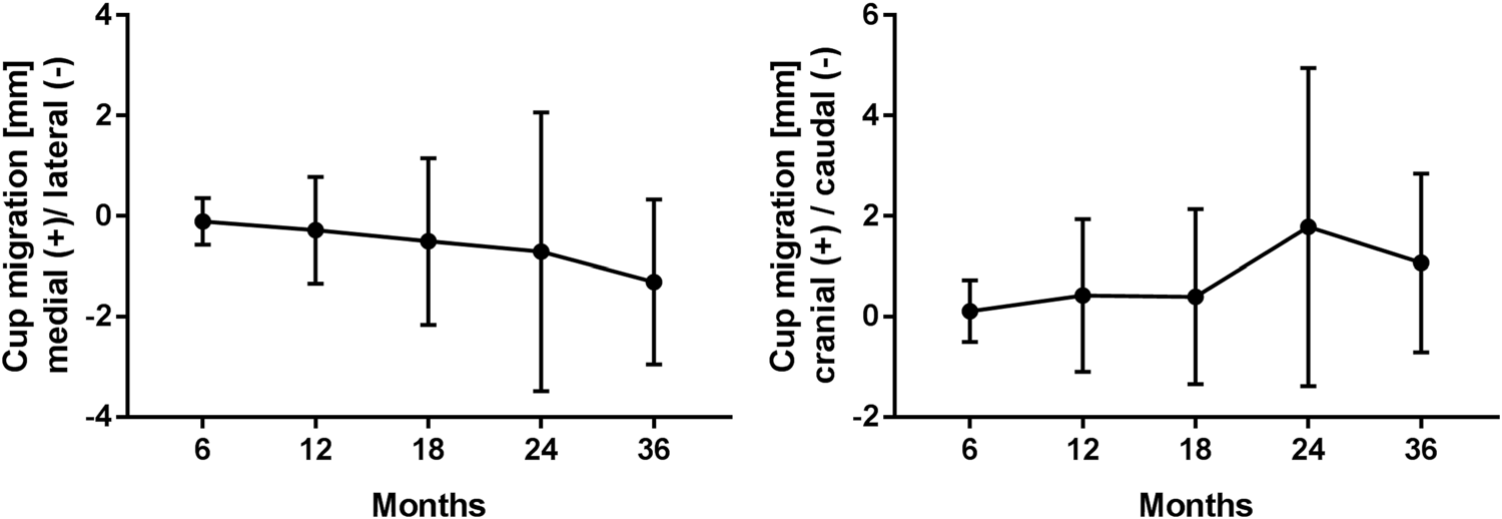
EBRA migration analysis results (medial/lateral and cranial/caudal) for the reconstruction cage

The inclusion criteria for the EBRA migration analysis were fulfilled in 45 of the 64 cases. A median of five x-rays per patient (range: 4–14) was assessed. Migration analysis showed a mean cup migration (medial and cranial) of 0.7 mm (range: 5.7 mm medial–9.6 mm lateral) and 1.8 mm (range: 1.7 mm caudal–12.6 mm cranial) in 24 months after implantation. Within 24 months only two of the 45 implants showed cup migration > 5 mm. The most severe cup migration was found in a patient presenting with an acetabular defect grade III A according to Paprosky’s classification. During the entire observation period, a migration > 5 mm was observed in seven hips in our study group. These were distributed over the following periods: two hips between 0 and 1 year, two hips between 2 and 3 years, one hip between 4 and 5 years, one hip between 6 and 7 years and one hip between 7 and 8 years (Fig. 3).

During the above-mentioned follow-up period, only four of the 64 GAP II cages were revised. The revision was performed after 4, 6 and 31 months. These revisions were due to implant failure (broken hook; *n* = 2), infection (*n* = 1) or aseptic loosening (*n* = 1). In one further case, revision surgery would have been necessary because of aseptic loosening after 51 months, but the patient’s state of health did not allow a revision procedure. Overall, the Kaplan–Meier analysis showed an implant survival rate of 94% (CI 95%, 82–98) after 24 months.

In three cases, the liner had to be revised after two weeks, 12 and 52 months, whereby the acetabular restoration cage was left in place. Two patients showed recurrent luxations after revision surgery. A postoperative infection was treated with DAIR (debridement, antibiotics and implant retention), thus necessitating the third-liner revision.

During the investigated follow-up period, seven of the 64 GAP II restoration cages showed a hardware failure that did not require revision surgery. A broken hook occurred in five cups. At least one broken screw was found in the five cups; none of theses cups were revised. The broken screw fixed the lateral flange of the cup to the ileum in a horizontal direction.

## Discussion

Revision THA combined with a severe acetabular defect is a complex and challenging procedure [27]. A wide range of treatment options is currently available, including the use of so-called restoration cages [28]. The purpose of the study was to perform a migration analysis of the GAP II reconstruction cage. In most cases, the GAP II cage is used with bone impaction grafting for management of acetabular defect during revision THA [3, 5, 10]. This is the first report on migration analysis of a reconstruction cage in revision THA. We also validated EBRA for migration analysis of reconstruction cages in revision THA. To the best of our knowledge, there are no published studies to date that evaluate EBRA or RSA (roentgen stereophotogrammetric analysis) in a revision setting [3, 5, 10].

Clinically published data show controversial success rates ranging from 60 to 100% after implantation of the GAP II cup [3, 5, 10] Inconsistency of implant survival can be related to the implant itself, as well as to the surgeon, the surgical technique, the chosen outcome assessments, statistical analysis, or the quality of the published study designs [3, 5, 10]. Therefore, objective parameters like migration analysis (e.g. EBRA) or RSA should be applied.

In our study, an average of 0.7 mm medial and 1.8 mm cranial cup migration was observed two years after surgery. In their publication Buttaro et al. prescribed an average medial and cranial migration of 1.4 mm and 2.2 mm [3]. Hosny et al. measured a mean migration of 1.9 mm medial and 2.1 mm cranial in their study according to the method by Nunn et al. [5, 29]. Both studies show greater cup migration than we found in our results. Several methods have been described to determine the migration of the acetabular component [21, 29–31]. We used the more accurate and well established EBRA method [21]. EBRA provides results with an accuracy of ± 1 mm [21].

Phillips et al. [32] confirmed the high measurement accuracy of EBRA by comparison to that of other methods. One method for migration measurement of acetabular components is the RSA [33]. The precondition for this is the perioperative implantation of tantalum balls in the pelvic bone near the acetabular cup. Two radiographs taken from different angles are needed for migration analysis. These x rays of the pelvis are taken in a special calibration cage in order to enable 3-dimensional evaluation with the appropriate software for measuring implant migration. As tantalum balls must be implanted, this type of migration analysis involves a major effort and cannot be performed retrospectively [30, 32, 33]. Abraham showed that EBRA can accurately measure migration of uncemented acetabular components used at revision THA [34] and that EBRA cup and RSA measurements had a good agreement on the classification of components above and below 1 mm at 2 years with a sensitivity and specificity of 100% and 87%, respectively [34]. In cases of pelvic discontinuity and the use of augments and cages, the accuracy of EBRA migration measurements was significantly poorer [34]. However, Abraham published only three cases, where a cage was used as a “cup‐cage construct” with or without an augment [34]. Our cohort consisted only of reconstruction cages used with bone graft and the number of investigated cases is 64, representing the largest series in the literature to date.

Duffy et al. [10] presented a first small study including 17 hips and a 58% success rate for the implant at a mean follow-up of 60 months. In their study, five implants had to be revised within 60 months, with four of the five revisions based on bone graft resorption [10]. We believe graft resorption was a cause of stress shielding and no bearing led to micromotion or played a major role in these failures [3]. The absence of structural bone may also explain these fatigue failures [3]. Nevertheless, Duffy et al. [10] recommended in their study that this implant be used only in the presence of a sufficiently stable bone stock situation. Buttaro et al. [3] abandoned further use of the GAP II restoration cage. In their study, nine of their 24 GAP II cages failed at a mean follow-up of 34 months, with six of the nine failed implants showing septic loosening [3]. In the study by Buttaro et al. the bone stock was replenished using bone impaction grafting according to the method described by Sloof et al. [3, 24]. Contrary to these results, Hosny et al. [5] observed a success rate of 100% at a mean follow-up of 49 months. A stable implant position is a precondition for the incorporation of the bone graft and bony ingrowth of the restoration cage [35, 36]. Otherwise, early migration of the implant leads to lysis of the bone graft, aseptic loosening and thus failure of the implant may occur [37].

This study has several limitations. First, it was a retrospective study without a control group. As a result, no statistical comparison of outcomes or cup migration with a control group using other implants was possible. Second, our study cohort was small. Still, these were not common cases, and even in high-volume centers the numbers were relatively small. Nevertheless, as far as we know, this is currently the largest study cohort in the literature using the GAP II in revision surgery. Third, we did not perform radiostereometric studies, which would likely detect earlier migration and perhaps predict some failures among those patients, who were functioning well at the time of the latest follow-up.

In summary, reinforcement rings and bone allografts have been proposed for the management of severe acetabular bone defects in revision hip surgery. The GAP II reconstruction cage showed in combination with bone impaction grafting low migration and revision rates at a mean follow-up of 35 months. In our study, the GAP II cage seems to be a good therapeutic option for revisions in THA with a severe acetabular defect. In addition, our results of the validation in an anatomical phantom model of the reconstruction cage revealed that EBRA cup analysis can now also be applied for reconstruction cages in revision THA. This will significantly increase the use of EBRA in revision arthroplasty and be an objective tool in the outcome and migration analysis.

Continued observation is necessary in order to observe the long-term success rates and follow radiologically conspicuous implants. Overall, precise preoperative evaluation of the patient as well as a patient-specific decision on the surgical procedure and the used implant is always required to ensure good mechanical stability of the implant and functionality of THA.

## Data Availability

Data will be sent if necessary.
